# Characteristics of circulating small noncoding RNAs in plasma and serum during human aging

**DOI:** 10.1002/agm2.12241

**Published:** 2023-02-22

**Authors:** Ping Xiao, Zhangyue Shi, Chenang Liu, Darren E. Hagen

**Affiliations:** ^1^ Department of Animal and Food Sciences Oklahoma State University Stillwater Oklahoma USA; ^2^ School of Industrial Engineering and Management Oklahoma State University Stillwater Oklahoma USA

**Keywords:** aging clock, circulating sncRNAs, human aging, machine learning

## Abstract

**Objective:**

Aging is a complicated process that triggers age‐related disease susceptibility through intercellular communication in the microenvironment. While the classic secretome of senescence‐associated secretory phenotype (SASP) including soluble factors, growth factors, and extracellular matrix remodeling enzymes are known to impact tissue homeostasis during the aging process, the effects of novel SASP components, extracellular small noncoding RNAs (sncRNAs), on human aging are not well established.

**Methods:**

Here, by utilizing 446 small RNA‐seq samples from plasma and serum of healthy donors found in the Extracellular RNA (exRNA) Atlas data repository, we correlated linear and nonlinear features between circulating sncRNAs expression and age by the maximal information coefficient (MIC) relationship determination. Age predictors were generated by ensemble machine learning methods (Adaptive Boosting, Gradient Boosting, and Random Forest) and core age‐related sncRNAs were determined through weighted coefficients in machine learning models. Functional investigation was performed via target prediction of age‐related miRNAs.

**Results:**

We observed the number of highly expressed transfer RNAs (tRNAs) and microRNAs (miRNAs) showed positive and negative associations with age respectively. Two‐variable (sncRNA expression and individual age) relationships were detected by MIC and sncRNAs‐based age predictors were established, resulting in a forecast performance where all *R* ^2^ values were greater than 0.96 and root‐mean‐square errors (RMSE) were less than 3.7 years in three ensemble machine learning methods. Furthermore, important age‐related sncRNAs were identified based on modeling and the biological pathways of age‐related miRNAs were characterized by their predicted targets, including multiple pathways in intercellular communication, cancer and immune regulation.

**Conclusion:**

In summary, this study provides valuable insights into circulating sncRNAs expression dynamics during human aging and may lead to advanced understanding of age‐related sncRNAs functions with further elucidation.

## Graphical abstract

Human aging is associated with increased susceptibility to age‐related diseases due to alteration of biological processes. Here we identified changes in extracellular small noncoding RNA (sncRNA) expression with age from plasma and serum samples. A machine learning‐based aging clock was developed using age‐related sncRNAs and is capable of predicting individual age information. As a result of profiling the circulating sncRNA transcriptome we identified putative core biomarkers linked to the aging process.

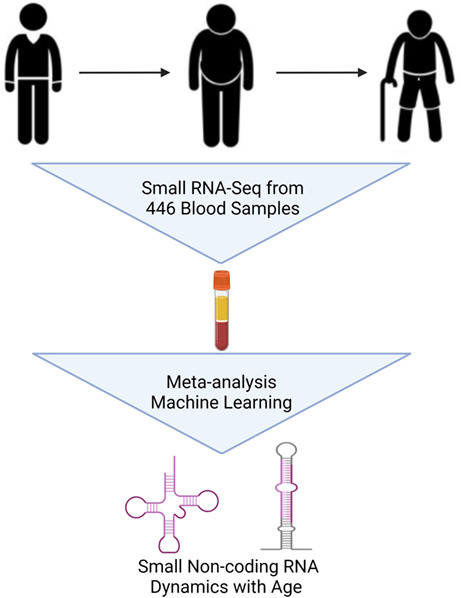

## 1 INTRODUCTION

Heterogeneity of human lifespan and health outcomes occurs due to differential aging process.^1^, ^2^, ^3^ Organismal aging is often accompanied by dysregulation of numerous cellular and molecular processes that triggers age‐related pathologies such as tissue degradation,^4^ tissue fibrosis,^5^ arthritis,^6^ renal dysfunction,^7^ diabetes,^8^ and cancer.^9^ The highly proactive secretome from senescent cells, termed the senescence‐associated secretory phenotype (SASP), is one of main drivers that cause age‐related pathogenesis through intercellular communication.^10^ The classical SASP includes secretome of soluble factors, growth factors, and extracellular matrix remodeling enzymes,^11^ and it can transmit age‐related information to the healthy cells via cell‐to‐cell contact.

As one of the emerging SASP components protected by extracellular vesicles (EVs), ribonucleoprotein (RNP) complexes, and lipoproteins,^12^ extracellular RNAs (exRNAs) are found in many biological fluids^13^ and can bridge the communication between “donor” and “recipient” cells through endocytosis, inducing paracrine senescence and pro‐tumorigenic processes.^14^, ^15^ Deep sequencing of human plasma exRNA revealed more than 80% of sequencing reads mapped to small noncoding RNAs (sncRNAs) in human genome, including microRNAs (miRNAs), PIWI‐interacting RNAs (piRNAs), transfer RNAs (tRNAs), small nuclear RNAs (snRNAs), and small nucleolar RNAs (snoRNAs).^16^ Extracellular miRNA expression in plasma of mice changes with age and cellular senescence can affect age‐related homeostasis throughout the body by circulating miRNA.^17^ Other studies uncovered the roles of circulating miRNAs in age‐related dysfunction such as osteogenesis imperfecta,^18^ decreased myelination,^19^ tumorigenesis,^20^ and cardiovascular disease.^21^ However, the molecular function of other circulating sncRNAs in aging and age‐related diseases has been overlooked, and their expression profiles during human aging process must be further characterized.

In this study, we determined the extracellular sncRNAs landscape during healthy human aging. Furthermore we generated an aging clock based on dynamic changes in extracellular sncRNAs and identified putative core sncRNAs with larger contribution weights in machine learning models for age‐related risks prediction. To achieve this, we used 446 pre‐selected small RNA‐seq data from plasma and serum samples (age: 20–99 years) and employed differential expression analysis and linear or nonlinear association measurements to determine age‐related sncRNAs as primary inputs for comprehensive machine learning modeling. Based on supervised machine learning models, aging estimators were created in high accuracy and sncRNAs candidates with top importance values in built models were considered as final age‐related biomarkers. Additionally, pathway enrichment of targets of core miRNAs strengthens our viewpoint that extracellular sncRNAs change with age‐related processes.

## 2 RESULTS

### 2.1 Overview of integrated human small RNAs dataset

**FIGURE 1:**
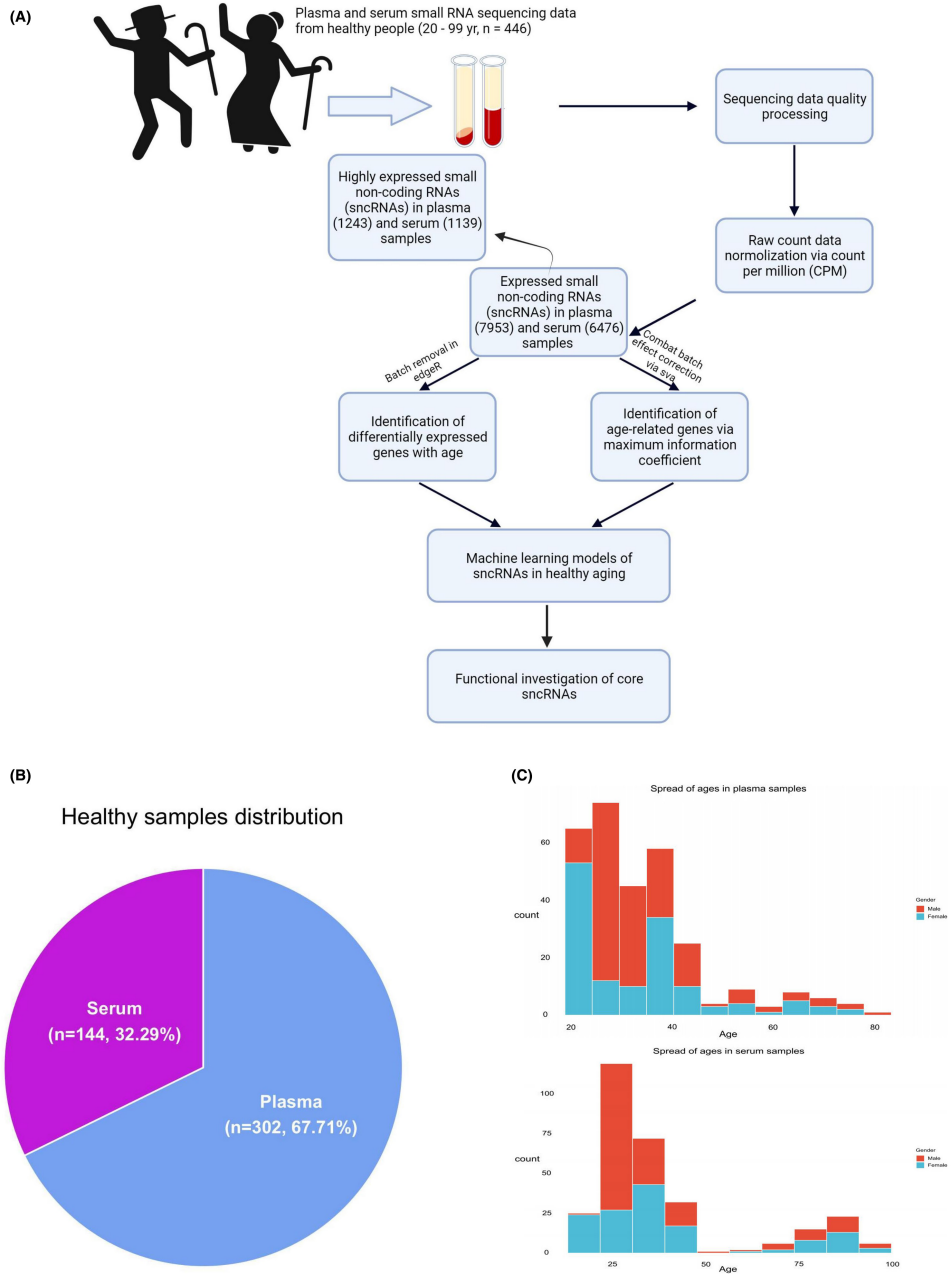
Identifying practical computational models of healthy aging via plasma and serum small noncoding RNAs (sncRNAs). (A) Flow chart of data preprocessing, normalizing, batch effect correcting, and analyses of 446 blood samples. (B) Proportion of plasma and serum samples from healthy donors. (C) Distribution of age and gender in plasma and serum

To profile sncRNAs features during human healthy aging, we obtained small RNA‐seq datasets from the Extracellular RNA (exRNA) Atlas data repository (https://exrna‐atlas.org).^22^ This work includes the studies for which information on age, health status, and gender, but only individuals having healthy aging process were retained for analysis. For datasets meeting the quality control standards established by the Extracellular RNA Communication Consortium (ERCC) (see experimental procedures), we created a bioinformatics procedure for reads mapping, processing, normalizing, categorizing, and modeling (Figure 1A). As a result of these criteria, 302 plasma and 144 serum samples (Figure 1B) were used in this study, with a similar number of samples representing each gender ranging from 20–99 years old (Figure 1C, Table S1). As these datasets originate from distinct studies with multiple sampling and library preparations, there are clear batch effects after Counts Per Million (CPM) normalization (Figure S1A,B). The ComBat function from the R package sva (v3.40.0) in Bioconductor^23^ was employed to reduce or eliminate batch effect that may deviate from actual cross‐study results (Figure S1C,D). These corrected data were used for correlation measurements and machine learning training described below.

### 2.2 Identification of expressed sncRNAs in plasma and serum

**FIGURE 2:**
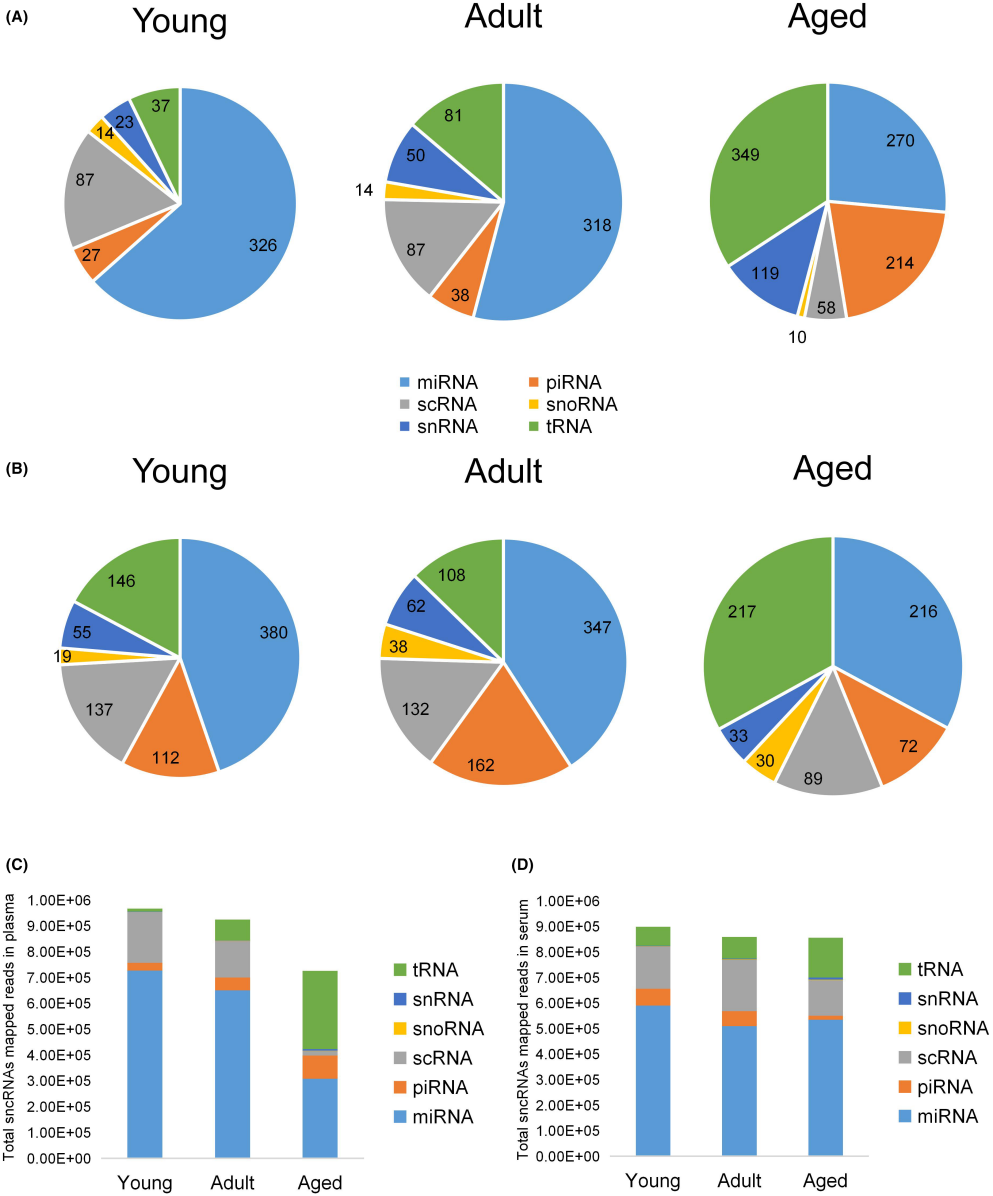
Highly expressed sncRNAs in plasma and serum. Subtype distribution of highly expressed sncRNAs, which meet the expression cutoff (≥10 CPM in ≥30% of samples) among young (20–30 years), adult (31–60 years), and aged individuals (≥61 years) in plasma (A) and serum (B). Total sequencing reads of highly expressed sncRNAs among three age groups in plasma (C) and serum (D)

To determine sncRNAs expressed during aging, we considered sncRNAs with ≥1 CPM in at least 30% of individuals within an age group (young (20–30), adult (31–60), and aged (61+) groups) as expressed sncRNAs. As a result, there were 7953 and 6476 sncRNAs observed in plasma and serum samples respectively (Figure 1A). Further, we identified highly expressed sncRNAs by increasing minimal CPM to 10, resulting in 1243 and 1139 sncRNAs retained in plasma and serum samples respectively (Figure 1A, Table S2). In terms of distribution of sncRNAs subtypes in three age groups, miRNAs account for a high proportion (26.5%–63.4%) of all sncRNAs in both plasma and serum, and their abundance consistently decreased with age (Figure 2A,B). tRNAs increased and became the dominant sncRNA in aged group while expression of miRNAs were reduced in older individuals (Figure 2A,B). The corresponding mapped reads are proportional to the number of each highly expressed subtype, even though miRNA showed relatively more sequencing reads than others in both plasma and serum (Figure 2C,D).

### 2.3 Exploring the correlation between sncRNAs and human aging

**FIGURE 3:**
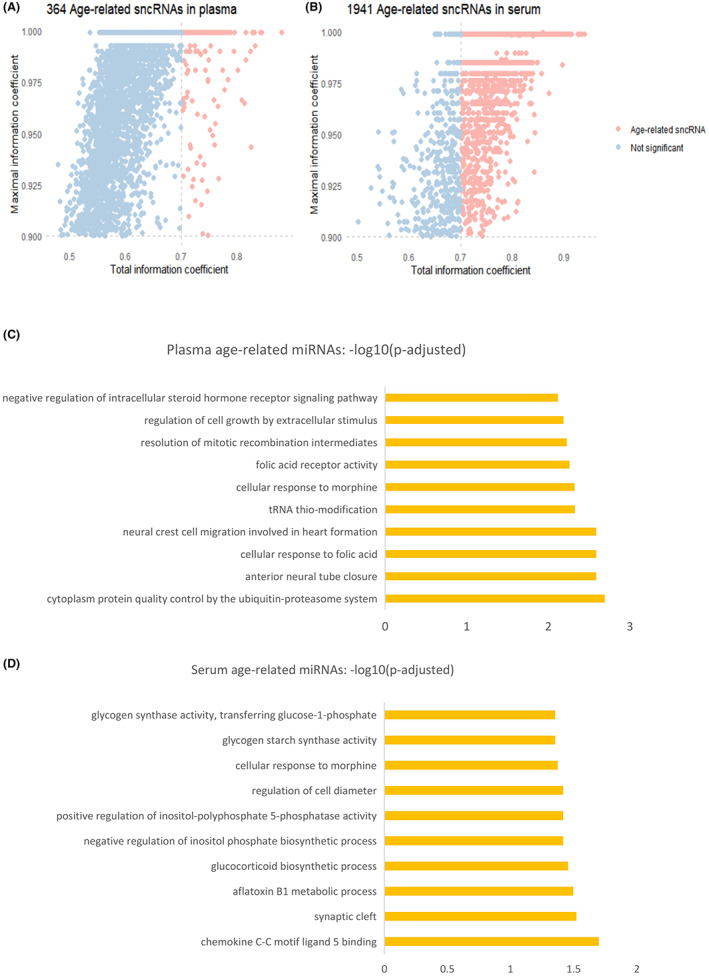
Identification of age‐related sncRNAs. MIC‐based age‐related sncRNAs in plasma (A) and serum (B), identified by both MIC and total information coefficient (TIC) values ≥0.7. Over‐representation analysis of biological process of MIC‐based age‐associated miRNAs targets in plasma (C) and serum (D) (*p*‐adjusted value <0.05)

We calculated the maximum information coefficient (MIC) (D. N.^24^) to investigate both linear and nonlinear associations between sncRNAs expression and corresponding individual age. By employing batch‐corrected data of expressed sncRNAs, we identified 364 and 1941 age‐related sncRNAs from plasma and serum respectively (Figure 3A,B, Table S3). Intriguingly, piRNAs became the most abundant sncRNAs in MIC measurement, with the number of snRNAs representing the second largest (Figure S2A,B). Similarly, the over‐represented biological processes of miRNA targets were identified, and cellular response and epigenetic modification were enriched in plasma (Figure 3C), while biosynthetic processes were significantly observed in serum samples (Figure 3D).

### 2.4 Core feature selection of age‐related sncRNAs

**FIGURE 4:**
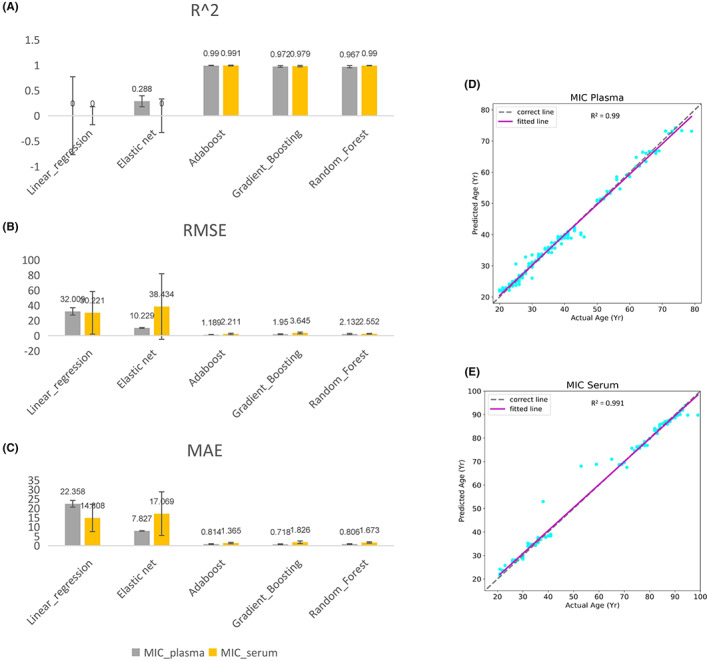
Performance evaluation of sncRNAs based aging clocks built by linear regression, elastic net, Adaptive Boosting, Gradient Boosting, and Random Forest approaches. Summary of *R* ^2^ value (A), root mean squared error (RMSE) (B), and mean absolute error (MAE) (C). (D) Model fit based on plasma MIC‐based associated sncRNAs. (E) Model fit based on serum MIC‐based associated sncRNAs. All model fits were constructed using Adaptive Boosting method.

As the expression of sncRNAs changes with age, further data‐driven analysis was conducted to construct a human aging clock. MIC‐based age‐correlated sncRNAs were used as inputs to train regression models in plasma and serum samples. Compared to the linear models, such as Linear Regression (without feature selection) and Elastic Net (feature selection through regularization), the tree‐based ensemble machine learning methods (including Adaptive Boosting, Gradient Boosting, and Random Forest regressors) showed stronger power of prediction with better performance in accuracy (Figure 4) since its great capability of learning the underlying nonlinear patterns. With stably ideal performance in test subsets (Table S4), all models inputting age‐correlated sncRNAs (MIC_plasma and MIC_serum) accurately predicted the ages of corresponding individuals in test sets, with average *R* ^2^ values greater than 0.96, root mean squared error (RMSE) values less than 3.7 years and mean absolute error (MAE) values less than 1.9 years (Figure 4A–C).

Due to the strong generalization ability in all ensemble learning methods, core sncRNAs associated with aging processes were determined by combined statistics and sum of importance ranks in the three methods was used as the criteria for core sncRNAs identification. As a result, there were 222 and 321 core sncRNAs overlapped in all three methods with MIC_plasma and MIC_serum as the inputs respectively (Table S5). Particularly, four snRNAs, three piRNAs, two small cytoplasmic RNAs, and one miRNA were identified as top core sncRNAs in plasma (Table 1). In serum samples, seven snRNAs, two tRNAs, and one small cytoplasmic RNA identified as top core sncRNAs in serum samples (Table 2).

**TABLE 1:** Top core sncRNAs associated with age in plasma

Model input	Gene name	RNA type	Adaboost	GB	RF	Sum of rank
MIC_plasma	piR‐33,748	piRNA	3	1	1	5
MIC_plasma	U5‐L97	snRNA	1	2	2	5
MIC_plasma	HY3‐L319	scRNA	2	6	3	11
MIC_plasma	U6‐L1016	snRNA	7	7	4	18
MIC_plasma	hsa‐miR‐11,181‐3p	miRNA	6	13	14	33
MIC_plasma	HY1‐L12	scRNA	22	4	10	36
MIC_plasma	piR‐61,840‐L3	piRNA	16	3	18	37
MIC_plasma	U7‐L212	snRNA	18	17	11	46
MIC_plasma	piR‐49,811	piRNA	20	8	23	51
MIC_plasma	U1‐L72	snRNA	36	5	26	67

*Note*: Importance ranking from three ensemble learning methods and corresponding sum of rank.

Abbreviations: Adaboost, Adaptive Boosting; GB, Gradient Boosting; RF, Random Forest.

**TABLE 2:** Top core sncRNAs associated with age in Serum

Model input	Gene name	RNA type	Adaboost	GB	RF	Sum of rank
MIC_serum	U5‐L192	snRNA	2	9	6	17
MIC_serum	U6‐L317	snRNA	27	4	4	35
MIC_serum	HY3‐L199	scRNA	4	5	36	45
MIC_serum	U2‐L87	snRNA	34	13	28	75
MIC_serum	U3‐L6	snRNA	16	56	24	96
MIC_serum	tRNA‐Thr‐AGT‐1‐1	tRNA	96	18	2	116
MIC_serum	tRNA‐Ala‐AGC‐8‐1‐tRF5	tRNA	37	44	71	152
MIC_serum	U2‐L1053	snRNA	135	3	15	153
MIC_serum	U3‐L119	snRNA	109	41	3	153
MIC_serum	U6‐L1640	snRNA	136	10	13	159

*Note*: Importance ranking from three ensemble learning methods and corresponding sum of rank.

Abbreviations: Adaboost, Adaptive Boosting; GB, Gradient Boosting; RF, Random Forest.

Notably, we also observed a gender‐specific model performance. When male‐only samples were used as training set for predicting female‐only test sets or vice versa, there were core sncRNAs unique to one gender (Figure S3A,B and Table S6), with slightly lower performance in *R* ^2^ and RMSE values compared to the models trained in gender‐mixed data (Figure S3C,D).

### 2.5 Core miRNAs are involved in aging‐related processes

**FIGURE 5:**
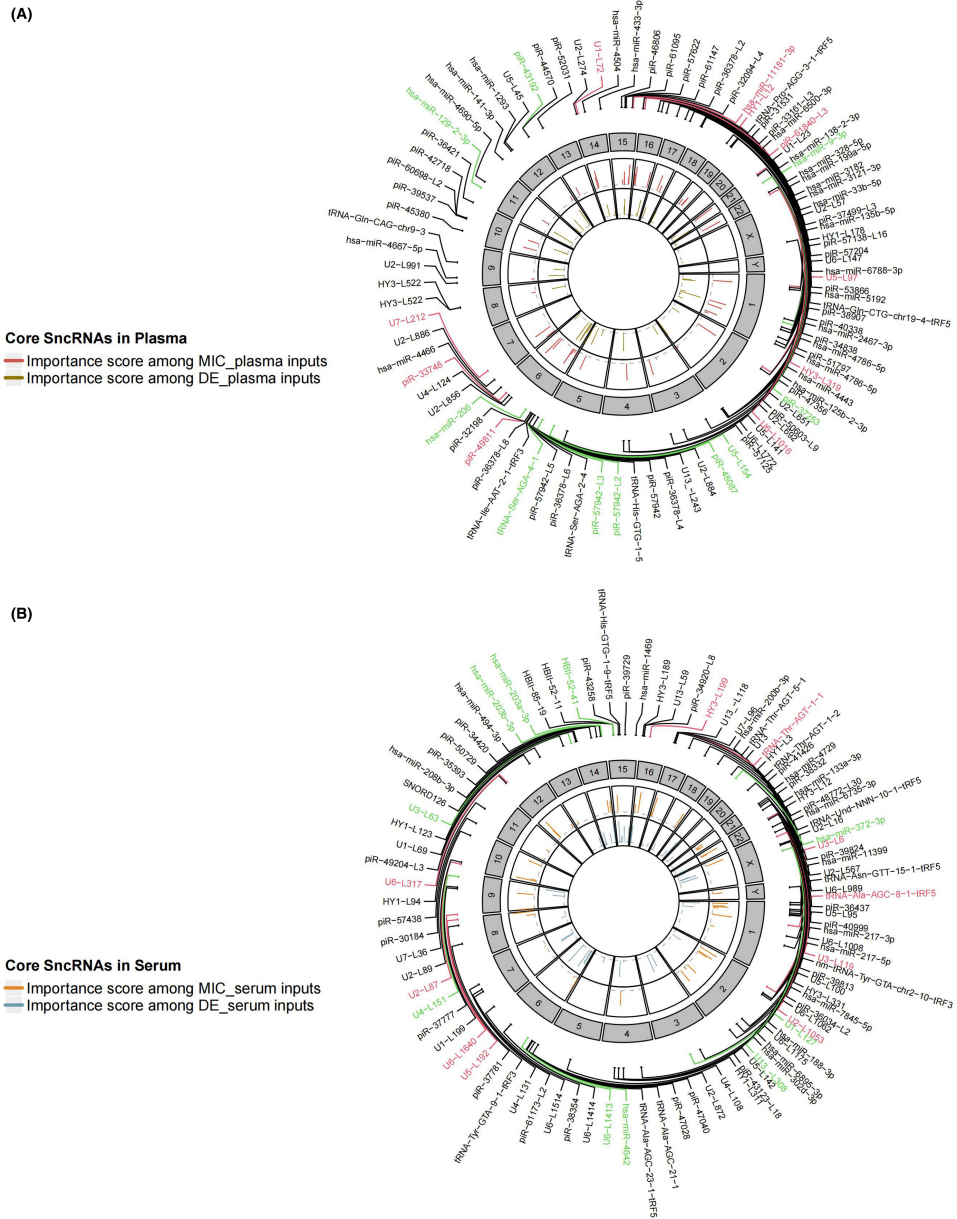
Top core miRNAs are associated with human aging and aging‐related disease. (A) KEGG pathway enrichment analysis of core miRNA targets. Pathway terms are ranked by combined score in Erichr.^73^ (B) Interaction network among core miRNAs (in red), targets (in blue), and corresponding regulatory proteins (in purple). Only targets and interacted proteins have validated function in cell senescence, human aging, and longevity (information from HAGR) are shown

To gain further insight into extracellular sncRNAs potential functions in a microenvironment, we focused on miRNAs, which are well characterized in post‐transcriptional gene regulation. The most ranked miRNA with the largest importance score in plasma and serum, hsa‐miR‐11,181‐3p and has‐miR‐7845‐5p (Table S5), were selected and their targets were separately predicted via the integration of eight miRNAs databases. The expressional profile of these two miRNAs in three age groups is in Figure S4 and corresponding targets are included in Table S7. As expected, these miRNA targets are enriched in canonical cell–cell communication pathways such as Sulfur relay system and Endocytosis pathways, as well as Immune development, Asthma and Ras signaling pathways that closely related to immune dysfunction and tumorigenesis during aging process (Figure 5A).

We also investigated the association between miRNA targets and protein coding genes previously validated in the human aging process from Human Aging Genomic Resources (HAGR),^25^ and we found targets, including *DDIT3*, *HLA‐DQA1*, *PTK2B*, *TTR*, and *YWHAG*, were experimentally identified to be associated with cancer progression, senescence, aging, and longevity (Table S8). Based on protein–protein interaction enrichment analysis, these targets were demonstrated to have regulatory relationship with hallmark proteins, such as PIK3R1, STAT3, IL7R, and JAK2 (Figure 5B and Table S9), which have function in cancer, immune response, and intercellular transduction, bolstering the probability that other non‐miRNA sncRNAs also have functions in aging and aging‐related diseases.

## 3 DISCUSSION

Our study comprehensively profiled the relationship of extracellular sncRNAs with age in blood and built an aging clock of healthy individuals using sncRNAs linear and nonlinear correlated with age. Previously, age predictors were developed through DNA methylation sites,^26^ transcriptome expression,^27^, ^28^ repeat elements,^29^ microRNAs,^2^ and protein abundance.^30^ This study provides the first detailed analysis of relationship between circulating sncRNAs and age based on regression models and core sncRNAs whose expression changes with age, allowing reliable age prediction.

From previous human biofluids studies, differential composition of small RNA has been reported in multiple biofluids. Godoy et al.^31^ used 12 normal human biofluids including plasma and serum in their study and for mapping reads of corresponding RNA sequencing (RNA‐seq), miRNA showed relative high fraction (63.8906%, median) in adult plasma compared to serum (36.0154%, median). However, the percentage of tRNA mapped reads in serum increased (42.2067%, median) and became the most abundant RNA biotype, while median value was 0.7759% in adult plasma. One study determined the diversity of small RNA in different biofluids, and tRNA showed the largest percentage of mapped reads (39.7%) in serum compared to plasma (5.8%) and whole blood (2.1%).^32^ Also, in the Max et al. study,^33^ they characterized extracellular RNAs (exRNAs) from both plasma and serum samples of the same healthy volunteers, and interestingly they showed substantial differences of small RNA composition, with higher proportion of miRNA in plasma and more tRNA reads in serum. We have some serum and plasma samples from the same individuals (Table S1) and consistent results were observed (Figure 2). Max et al.^33^ also concluded that different biofluid types, even though they come from the same origin, plasma and serum show significant variable that impact exRNA profile. One of the reasons is that additional absorption and continuous degradation of exRNAs by retained blood clot will reduce exRNA abundance.^33^ So proper exRNA isolation is essential and immediate platelet and cell debris depletion for plasma collection may avoid losses of exRNA characteristics as much as possible.

It is of interest to identify a detectable increase of highly expressed tRNAs in aged individuals, and it has been reported that spleen and brain had the highest tRNA expression,^34^ which may indicate unique and differential biological process happen as individuals age. A previous report similarly finds tRNAs were the second most abundant sncRNAs in healthy adults (20–40 years) when small cytoplasmic RNA was not mentioned.^35^ Unlike tRNAs driving protein synthesis, tRNA‐derived small RNAs (tsRNAs), including tRNA‐derived fragment (tRF) and stress‐induced tRNA halves (tiRNA), have been uncovered as aging process related sncRNAs.^36^ Similar as human studies, the expression of tsRNAs increased during aging in *Drosophila*,^37^ *C. elegans*,^38^ and mouse brain cells.^39^ Compared with healthy controls, differential expression of tsRNAs in age‐related diseases has been employed in disease prediction such as Alzheimer's disease and Parkinson's disease,^40^ ischaemic stroke,^41^ and osteoporosis.^42^ tsRNAs have roles not only in potential biomarkers, but also in expressional regulation of age‐related mRNAs.^36^ For example, 5′‐tRF^Tyr^ from tyrosine pre‐tRNA can silence PKM2, which is the inhibitor of p53, to cause p53‐dependent neuronal death.^43^ The number of highly expressed miRNA in our study displayed a decreased tendency in older group, and it has been observed in both plasma and serum. Both core miRNAs identified by machine learning models were found to have reduced expression as age increased, similar to decreased expression of a majority of age‐associated miRNAs in whole‐blood,^2^ serum,^44^ and peripheral blood mononuclear cells.^45^

It has been previously demonstrated that circulating sncRNAs from serum samples show strong association with human aging,^46^ while the human aging modeling based on regression relationship was not yet built. In our study, potential function of core sncRNAs was predicted via miRNA target prediction, and these targets showed enrichment in cancer, cell cycle, and longevity regulating pathways. There are overlapping genes included in both cancer and longevity regulation pathways, and this result was consistent with early study that profiled miRNAs expression between young and old individuals.^45^ For example, increased *PIK3R1* expression has been identified to impair anti‐tumor effect through PI3K‐Akt activation in breast and ovarian cancer chemotherapy.^47^, ^48^ Previous research determined that protein level of p85α, which is the subunit of *PIK3R1*, was elevated with age, and age‐associated miRNAs that potentially target *PIK3R1* were downregulated.^45^ Studies in human aging also show that sequence variations within *PIK3R1* gene are significantly correlated with longevity,^49^ and individuals with different genotypes of *PIK3R1* were associated with longevity through reduced mortality risk in cardiovascular disease.^50^ Interestingly, both core miRNAs (hsa‐miR‐11,181‐3p and has‐miR‐7845‐5p) that are potentially involved in PIK3R1 regulation (Figure 5B) showed lower expression in aged individuals (Figure S4). The hsa‐miR‐11,181‐3p has been used as biomarker for identification of glioma brain tumors from other brain tumor types.^51^ By suppressing Wnt signaling inhibitor APC2, overexpression of hsa‐miR‐11,181‐3p can promote Wnt signaling pathway and increase cell viability in colon malignant tumor cell line.^52^ For has‐miR‐7845‐5p, its expression in serum has been applied in constructing diagnostic classifier of ovarian cancer,^53^ and higher expression was also observed in serum of patients with persistent atrial fibrillation.^54^ Some direct targets of core miRNAs have been determined as drivers of age‐related process. For example, protein tyrosine kinase 2β (PTK2B) is a tyrosine kinase activated by angiotensin II through Ca^2+^‐dependent pathways to mediate ion channels as well as map kinase signaling pathway.^55^ PTK2B is involved in cell growth, inflammatory response, and osmotic pressure regulation after activation and mutated PTK2B is statistically associated with hypertension in Japanese population.^56^ PTK2B has also been reported in memory formation and corresponding protein variants can trigger cognitive dysfunction and higher prevalence of Alzheimer's disease.^57^ As a nuclear protein that activated by DNA damage, DNA‐damage inducible transcript 3 (DDIT3) shows increased expression and prevents gene transcription by dimerizing with transcription factors.^58^ Specifically, DDIT3 plays role in endoplasmic reticulum (ER) protein processing and resulted ER stress promotes cardiomyocyte senescence in mouse hearts.^59^ The function of most of age‐associated sncRNAs identified in this study is unknown and further investigation into their function may provide meaningful results.

We also observed the mild sex‐dependent differences in the aging clock modeling. Similarly, a previous study indicated that sncRNAs differences between genders were minor^33^ and sex‐specific training sets have relatively low performance score in prediction compared to the gender‐mixed training sets. During this process, some gender‐dependent core sncRNAs were identified, including male‐specific sncRNAs piR‐31,143 and piR‐48,977 in plasma, male‐specific sncRNAs piR‐33,527 and piR‐57,256 in serum, female‐specific sncRNAs hsa‐miR‐3789 and U5‐L214 in plasma and female‐specific sncRNAs U6‐L989 and piR‐30,597 in serum (Table S6). Further mechanistic study is needed to uncover their prospective role in aging and aging‐related disease.

A major limitation of our current study is the corresponding datasets utilized were developed by researchers for different, unique projects and with multiple RNA extraction protocols, which may bias extracellular RNA abundance.^35^ Furthermore, trait information such as ethnicity, body mass, and smoking habits were not considered in our study due to the lack of information, and a more sophisticated and systematic sample processing and recording would help future research on big data‐based human aging modeling.

In conclusion, we provide a novel insight into the circulating sncRNAs profile of human aging. We developed predictive models in uncovering core sncRNAs and estimated age by utilizing meta‐analysis based correlation measurement and machine learning modeling. The sncRNA dynamics with age provide valuable references for extracellular RNA study in aging, and the potential mechanisms of age‐related intercellular communication by sncRNAs need further investigation.

## 4 EXPERIMENTAL PROCEDURES

### 4.1 Data acquisition and filtration

Human small RNA‐Seq datasets in the extracellular RNA (exRNA) Atlas data repository (https://exrna‐atlas.org)^22^ were queried with studies filtered using the following requirements: (1) data were sequenced from plasma/serum samples; (2) samples have definitive age and gender information within each study; and (3) the donor of corresponding samples should have a healthy status and was sampled as a control individual for the study. As a result, two studies (Accession ID: EXR‐MTEWA1ZR3Xg6‐AN and EXR‐TTUSC1gCrGDH‐AN) were included in both plasma and serum studies, and two studies (Accession ID: EXR‐TPATE1OqELFf‐AN and EXR‐KJENS1sPlvS2‐AN) were obtained with only plasma and serum samples respectively and 366 plasma and 188 serum samples passed preliminary filtration. To avoid genes' expressional bias due to the low sequencing reads and host genome contamination, we only retained samples that met the quality control (QC) standards developed by Extracellular RNA Communication Consortium (ERCC). Briefly, individual dataset should have a minimum of 100,000 reads that aligned to annotated RNA transcript (including miRNAs, piRNAs, tRNAs, snoRNAs, circular RNAs, protein coding genes, and long noncoding RNAs), and ratio of transcriptome reads over total sequencing reads should be more than 0.5. Consequently, 302 plasma and 144 serum samples (Table S1) were retained for further analysis.

### 4.2 Quantification and batch effect removal

To generate expression matrices of sncRNAs, read adaptors and low quality bases were removed using the Trim Galore (v0.6.5) wrapper.^60^ Clean reads were aligned and quantified with bowtie2 (v2.4.4)^61^ and samtools (v1.1.4)^62^ through miRNAs and other sncRNAs annotation file from miRBase (Release 22.1) and the DASHR (v2.0)^63^ database, respectively. The raw sncRNAs expression results were integrated and processed in R (v4.1.1) computational environment for identifying age‐related sncRNAs after preprocessing. To correct for actual expression characteristics masked by sequencing depth variability, gene read counts were transformed into CPM values after measuring normalized library sizes by edgeR (v3.14) package.^64^ Since there were still obvious batch effects observed via principal component analysis (Figure S1), we conducted batch removal using the ComBat function in sva package (v3.40.0)^23^ and processed CPM‐based data showed improved sample clustering by age (Figure S1). Batch‐effect corrected data were used for identifying maximum information coefficient and constructing machine learning models described below.

### 4.3 Identification of association between sncRNAs and age

To select the sncRNAs representative of the age prediction model, the maximal information coefficient (MIC),^24^ which permits the identification of important, difficult‐to‐detect associations,^65^ was used to identify and screen the linear or nonlinear correlations between each sncRNA expression (*X*) and the individual's chronological age (*Y*). Reshef et al.^24^ reported that MIC − ρ^2^ to be near zero for linear relationships and MIC − ρ^2^ > 0.2 for nonlinear relationships, where ρ^2^ is the coefficient of determination (*R* ^2^). We also employed total information coefficient (TIC) to evaluate the power of independence testing between *X* and *Y*.^66^ The sncRNAs having both MIC and TIC values greater than 0.7 with actual age were retained for building models.

### 4.4 Comprehensive machine learning modeling

The corrected expression data of sncRNAs selected from differential expression analysis and MIC‐based correlation measurement were used for machine learning modeling. Since sncRNAs expression inputs could be seen as the explanatory variable X, which is a high dimensional vector, the modeling process was performed as a regression analysis problem and was formularized as:

(1) $$y=\hat{f}\left(X\right)$$

where X denotes the sncRNA inputs, *y* denotes individual's age, and $\hat{f}$ denotes the fitted mapping function. Ensemble learning including Adaptive Boosting, Gradient Boosting, and Random Forest were leveraged in this study, taking advantage of their strong generalization ability achieved by multiple weak learners combination.^67^ Based on manual parameter tuning, the parameter “number of estimators,” which is the number of weak learners (i.e., the regression tree in this study) to be integrated in model fitting, was determined in each specific model based on the overall performance (RMSE, *R* ^2^, and MAE, showed in Table S10). The performance of ensemble learning is compared with linear regression and elastic net. The corresponding importance of each sncRNA was calculated as impurity‐based feature score (sum to 1), which can be used to determine the fraction of sncRNA that it makes contribution to distinguish.^68^ Potentially core sncRNAs were determined by sorting the corresponding sum of ranks of their importance values in each ensemble learning model.

Since the number of samples is different in each age group (young, adult, and aged), simple k‐fold cross‐validation may cause uneven sampling and then trigger bad model performance due to over‐fitting. Therefore, stratified k‐fold cross‐validation is a better option to avoid this issue by selecting approximately the same proportions of samples in each pre‐set age group to the training set (Figure S5). In this study, we stratified fivefold cross‐validation based on the overall sample size. The regression modeling was conducted under Python 3.8.8 and scikit‐learn 0.24.1.^69^

### 4.5 Targets prediction of age‐related miRNAs

To better understand the potential function of circulating sncRNAs changing with age, we primarily predicted the targets of miRNA candidates by using multiMiR R package (V3.14),^70^ which integrates eight microRNA‐target databases (DIANA‐microT, ElMMo, MicroCosm, miRanda, miRDB, PicTar, PITA, and TargetScan).

### 4.6 Functional enrichment analyses

Functional enrichment analyses of genes targeted by age‐related miRNAs performed through Enrichr gene list‐based enrichment analysis tool.^71^ We used the combined score, which is a combination of the *P* value and z‐score, to offset the false positive rate caused by the different length of each term and input sets. For direct miRNAs functional enrichment, an over‐representation analysis was performed via miRNA Enrichment Analysis and Annotation Tool (miEAA 2.0),^72^ with expressed miRNA sets as the background set and *P* values were adjusted using Benjamini‐Hochberg (BH) procedure.

## AUTHOR CONTRIBUTIONS

PX performed the experiments and contributed to project design, data collection, execution of machine learning modeling and analysis, and manuscript writing. ZS and CL contributed to experimental design and execution of machine learning modeling and analysis. DEH contributed to data collection, analysis, and manuscript writing.

## FUNDING INFORMATION

Not applicable. This research did not receive external funding.

## CONFLICT OF INTEREST

The authors have no conflicts of interest to declare.

## Supporting information


Figure S1.

Figure S2.

Figure S3.

Figure S4.

Figure S5.

Table S1.

Table S2.

Table S3.

Table S4.

Table S5.

Table S6.

Table S7.

Table S8.

Table S9.

Table S10.

## Data Availability

All of the small RNA‐Seq raw data (FASTQ) files and corresponding metadata are available directly from Extracellular RNA (exRNA) Atlas data repository with study ID (EXR‐MTEWA1ZR3Xg6‐AN, EXR‐TPATE1OqELFf‐AN, and EXR‐TTUSC1gCrGDH‐AN), or from the database of Genotypes and Phenotypes (dbGaP) with accession ID phs000727.v1.p1 for study EXR‐KJENS1sPlvS2‐AN.
