# Supplementary figures and images for: Characteristics of circulating small noncoding RNAs in plasma and serum during human aging
Source: Aging Med (Milton). 2023 Feb 22;6(1):35–48. doi: 10.1002/agm2.12241 (PMC10000275; doi:10.1002/agm2.12241)

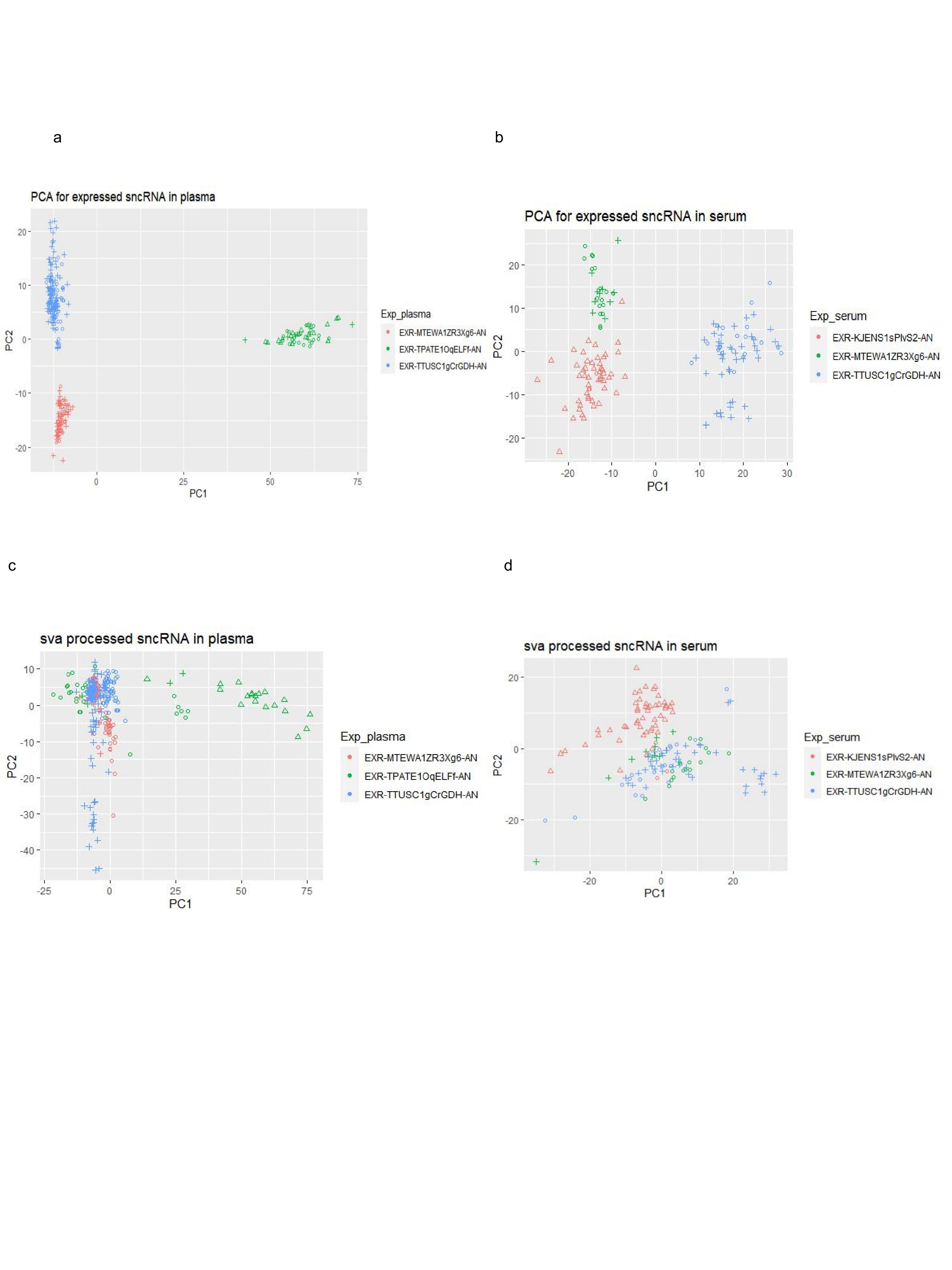

Supplement: Supplementary file 1 — Figure S1. [file AGM2-6-35-s009.jpg]

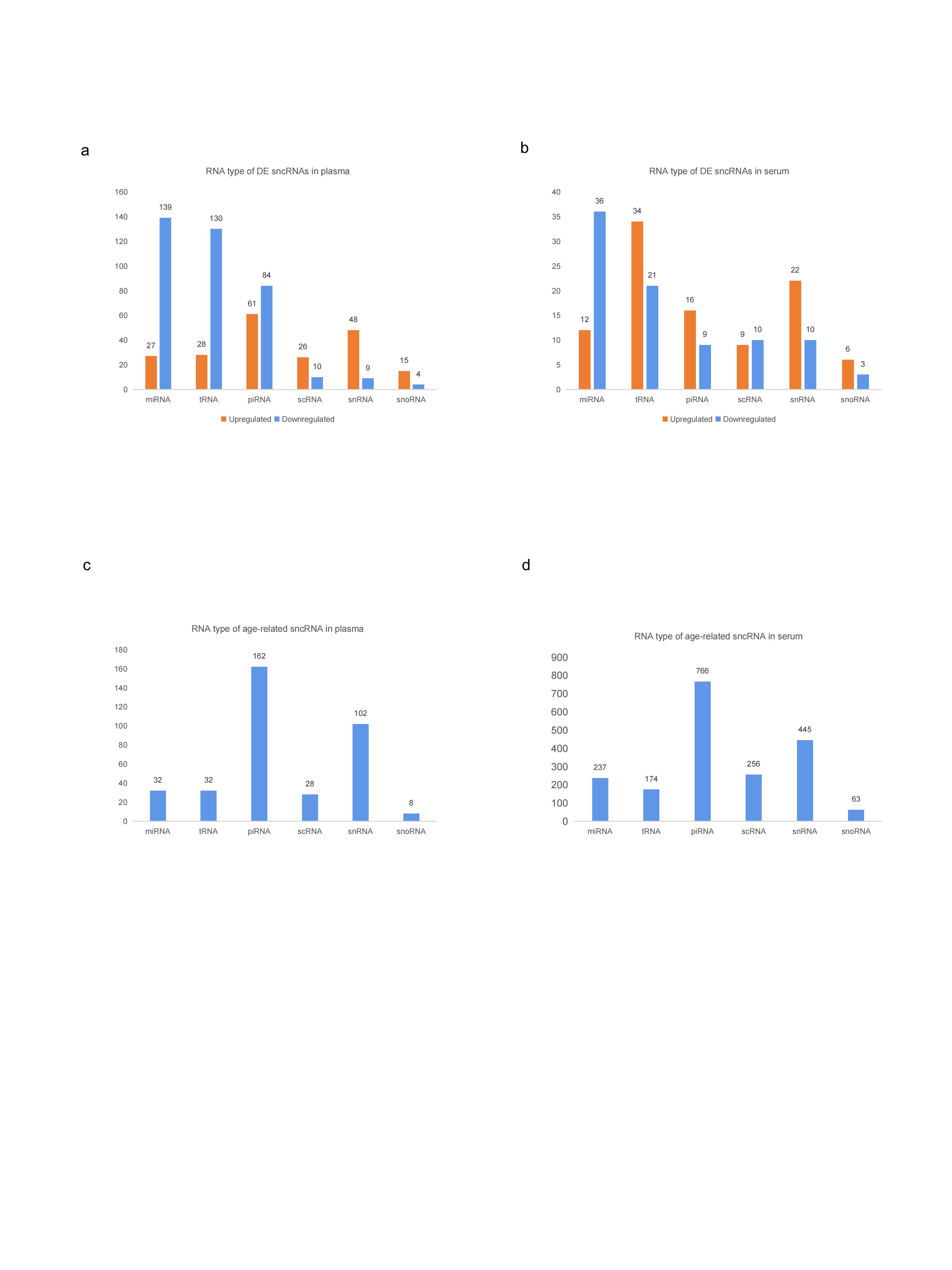

Supplement: Supplementary file 2 — Figure S2. [file AGM2-6-35-s013.jpg]

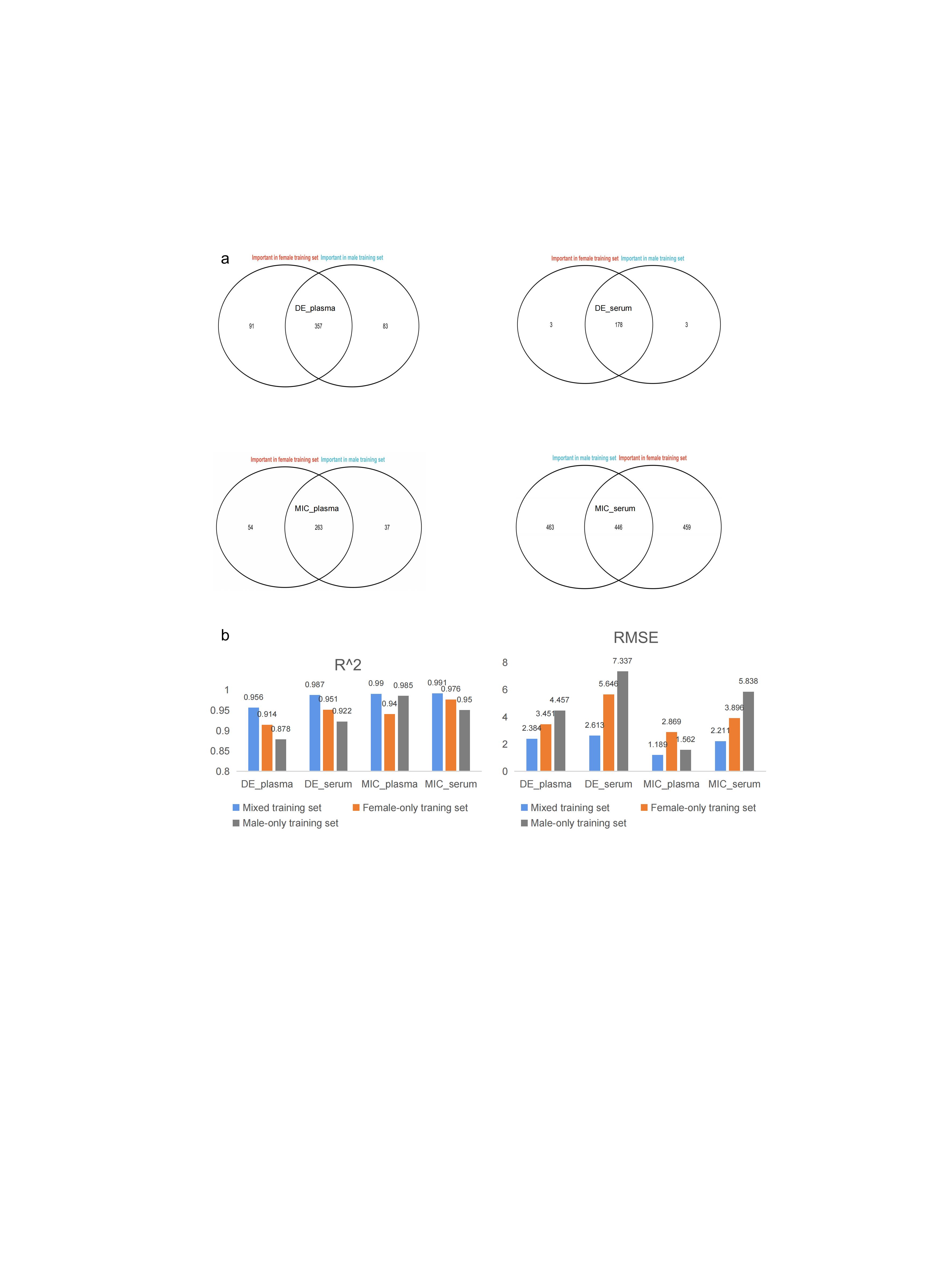

Supplement: Supplementary file 3 — Figure S3. [file AGM2-6-35-s003.jpg]

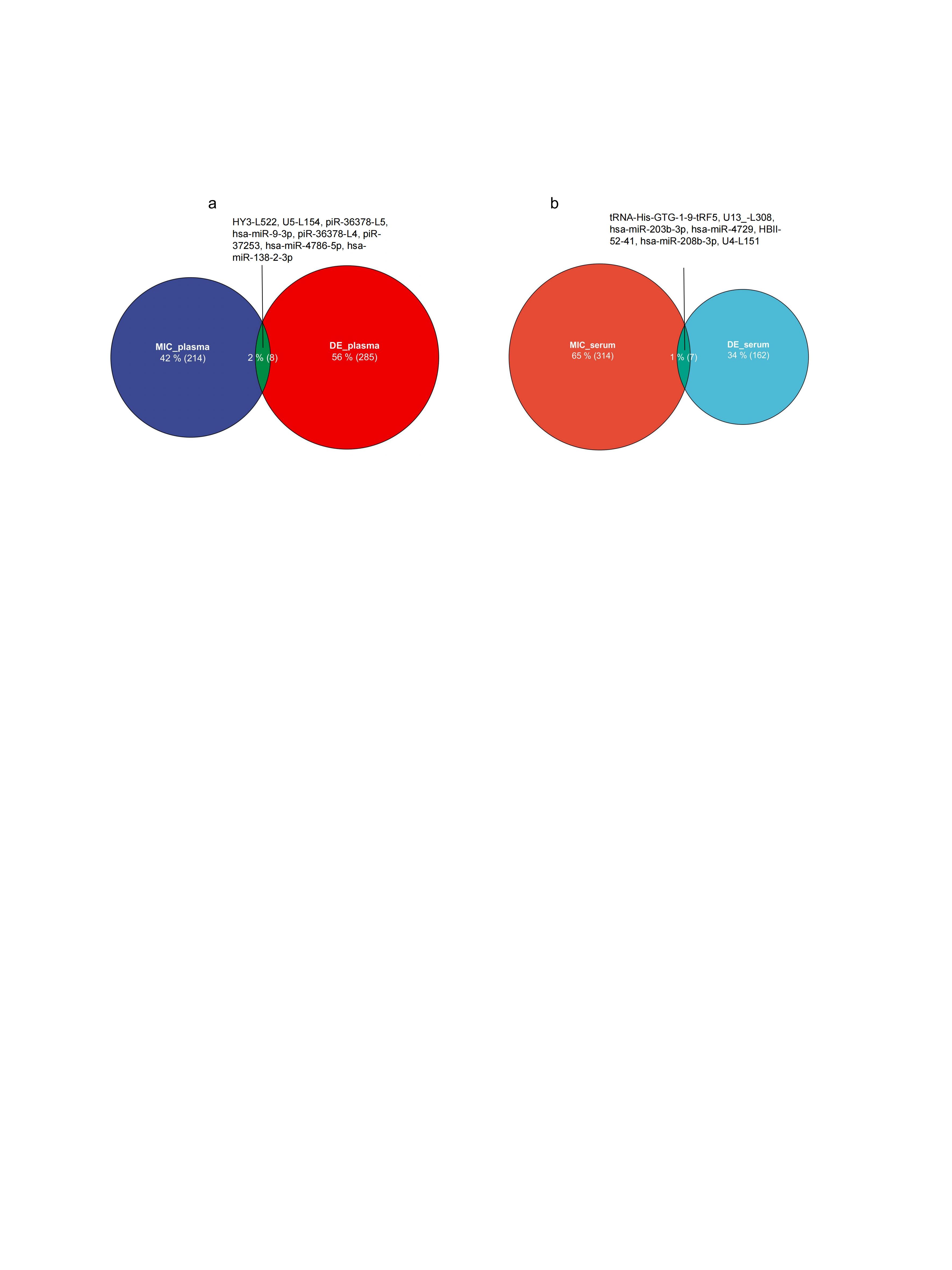

Supplement: Supplementary file 4 — Figure S4. [file AGM2-6-35-s012.jpg]

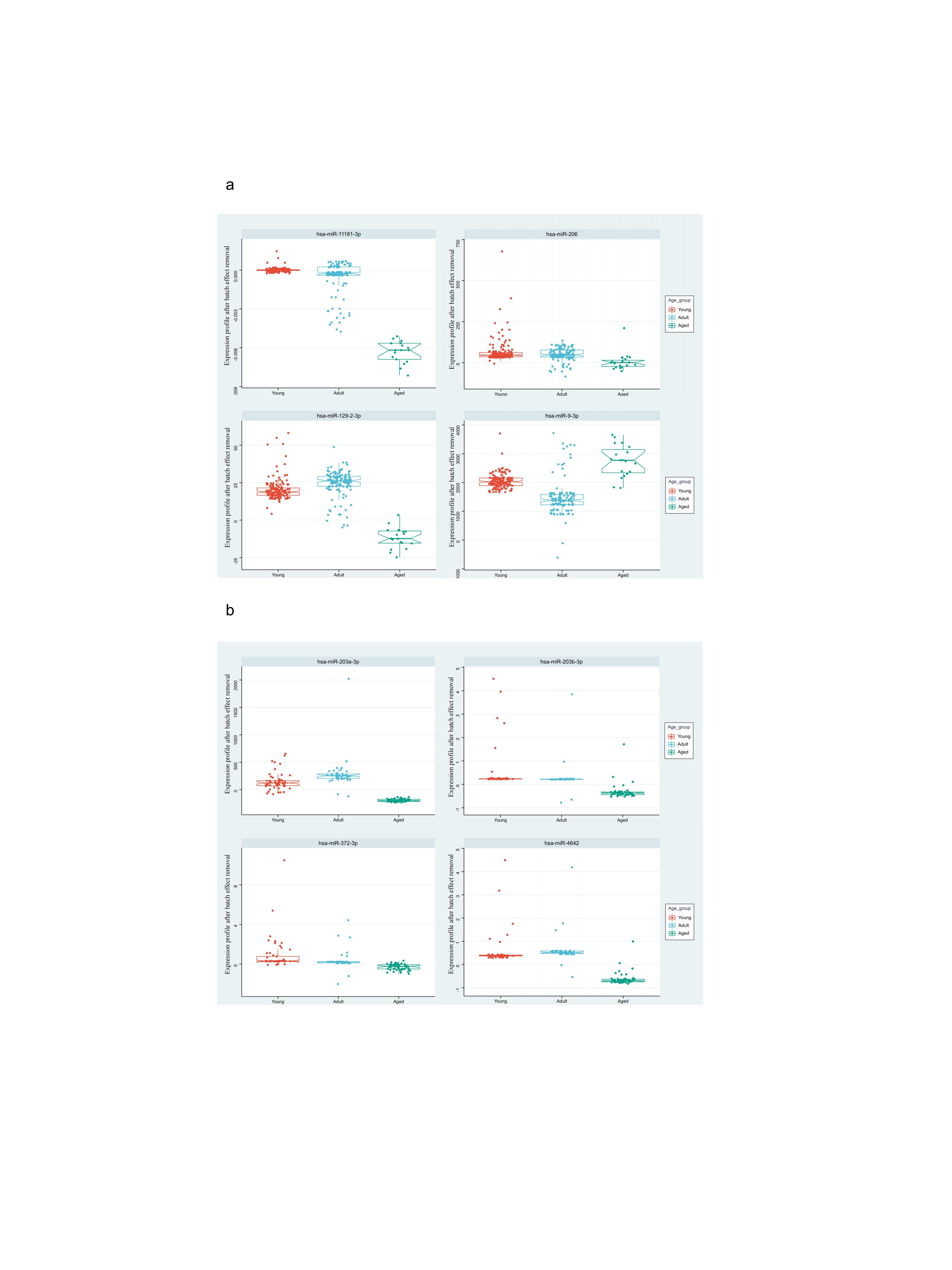

Supplement: Supplementary file 5 — Figure S5. [file AGM2-6-35-s005.jpg]
